# Multidimensional internet use related to cognitive performance in older persons: a nationwide cross-sectional study

**DOI:** 10.3389/fpubh.2024.1492331

**Published:** 2024-12-16

**Authors:** Yue Fan, Hua Wei, Qunshan Tao

**Affiliations:** School of Pharmaceutical Economics and Management, Anhui University of Traditional Chinese Medicine, Hefei, China

**Keywords:** multidimensional internet use, cognitive ability, older adults, CHARLS, PSM

## Abstract

**Introduction:**

The internet has been increasingly recognized as a potential driver for enhancing cognitive functioning in older adults; however, the mechanisms underlying this relationship remain insufficiently understood in the literature. This study aimed to investigate the associations between different dimensions of internet use—activity, device, and frequency—and cognitive performance in older adults.

**Methods:**

This cross-sectional study utilized data from the fourth wave of the China Health and Retirement Longitudinal Study (CHARLS), involving a nationally representative sample of 17,676 participants aged 60 years and above. Multiple linear regression analyses were conducted to explore the relationship between MIU and cognitive ability. To explore the moderating effect of age on the relationship between internet use and cognitive functioning, the population was divided into upper (over 75 years) and lower (under 75 years) age groups.

**Results:**

The analysis revealed a significant positive association between internet use and cognitive function. Specifically, MIU were found to contribute positively to cognitive performance. Subgroup analyses showed that participants aged 60–75 years benefited more from internet use, whereas those over 75 years exhibited a weaker association, indicating a potential decline in the cognitive benefits with advancing age.

**Conclusion:**

According to the results of the present study, MIU significantly increased the cognitive ability of older adult individuals. Additionally, MIU strongly influences components of cognitive functioning, including language, attention, calculation ability, orientation, memory. However, aging may weaken the relationship between MIU and cognitive ability.

## 1 Introduction

Cognitive impairment denotes a substantial decline in the lifelong learning process, encompassing areas such as quantitative reasoning, memory consolidation, and enhanced functional capacity. This decline may arise due to self-induced factors or external influences (1). Cognitive impairment represents an intermediate stage between normal aging and dementia and has emerged as a significant global concern in both developed and developing countries due to the growing aging population (2). Cognitive impairment primarily manifests as dementia, with its incidence and prevalence on the rise. Despite this, management costs remain high, and treatment outcomes continue to be suboptimal (3). Currently, around 50 million people worldwide are living with dementia, a number projected to rise to 152 million by 2050 (4). China has the largest number of patients with dementia, which places significant strain on public and healthcare systems (5). Therefore, in the absence of effective drugs and preventive measures, finding alternative methods to prevent cognitive impairment is essential.

Although this notion lacks clinical evidence (6), using the internet (a tool across the limitations of time and space) is believed to maintain and enhance the cognitive ability of older persons; for instance, internet searches may serve as a form of mental exercise, potentially strengthening neural circuits (7). Additionally, the internet can be used in many ways to provide a large amount of fresh information and remote contact with family and friends (8), which can promote cognitive functioning. Furthermore, internet use has been shown to have a positive effect on cognitive performance in older adults (9–12). In a cohort study, the risk of developing dementia was approximately half that of frequent internet users compared with infrequent internet users (13). This may be due to the process of searching for and processing information (14, 15). Moreover, other studies have shown that computer use contributes to improved cognitive performance, particularly memory (16). However, some scholars have expressed the diametrically opposite view that excessive internet use can lead to cognitive decline (17, 18), whereas an intervention study on computerized internet training revealed that excessive internet use had little effect on cognitive performance in older adults (19). Additionally, a meta-analysis indicated that technology use may lead to attention problems through repetitive attentional shifting and multitasking (20).

To date, several empirical studies have explored the relationship between internet use and cognitive ability in older adults. However, a review of the literature reveals several research gaps. First, most studies examine the relationship between internet usage and cognitive ability in broad terms, without exploring the specific impacts of different online activities, devices, and frequencies on cognitive functions. Second, while research on cognitive abilities often focuses on overall levels, few studies address the relationships between specific components of cognitive ability and internet use. Third, the findings from existing studies are inconsistent. Therefore, this study seeks to explore the relationship between multidimensional internet use (MIU) and cognitive ability in older adults, using data from the China Health and Retirement Longitudinal Study (CHARLS) developed by Peking University.

## 2 Materials and methods

### 2.1 Data processing

The CHARLS is a nationally representative longitudinal survey focused on the middle-aged and older population (aged 45 and above) (21). Led by Peking University in China, it was conducted in collaboration with the University of Oxford in the UK and the University of California, Berkeley, in the U.S. The main CHARLS consists of seven modules covering demographics, family background, health status, socioeconomic status, and the environment (community and county policy questionnaires). Since 2011, CHARLS has adopted multistage stratified PPS sampling and conducted face-to-face and computer-assisted interviews. The interviewers used electronic devices (such as tablets, laptops, or specialized handheld devices) to administer the survey questions directly to the participants.

Data from 2018 were used in this study. A total of 8,676 individuals were excluded because they were under 60 years old. After excluding 1,869 participants with missing values for independent variables and cognitive function scores, and an additional 2,509 individuals with missing control variable values, the final sample consisted of 6,762 participants. To control for potential confounders, a 1:1 propensity score matching (PSM) was applied based on whether or not individuals used the internet. Following matching, 606 participants were included in both the internet-use and non-internet-use groups (Tables 1, 2).

**Table 1:** Comparison of the two groups at baseline (mean ± SD/*n*, %).

Sociodemographic variables	Groups	Before PSM adjustment			*p*	After PSM adjustment			*p*
		Total (*n* = 6,762)	Internet use (*n* = 606)	Noninternet use (*n* = 6,156)		Total (*n* = 1,212)	Internet use (*n* = 606)	Noninternet use (*n* = 606)	
Age (years)		68.08 ± 0.10	65.90 ± 5.14	68.30 ± 6.14	<0.001	66.36 ± 5.66	65.90 ± 5.14	66.82 ± 6.11	0.005
Gender	Female	2,978 (44.04%)	233 (38.45%)	2,745 (44.59%)	0.004	449 (37.05%)	233 (38.45%)	216 (35.64%)	0.312
	Male	3,784 (55.96%)	373 (61.55%)	3,411 (55.42%)		763 (62.95%)	373 (61.55%)	390 (64.36%)	
Region	Rural	1,517 (22.55%)	338 (56.43%)	1,179 (19.24%)	<0.001	541 (45.08%)	338 (56.43%)	203 (33.78%)	<0.001
	Urban	5,210 (77.45%)	261 (43.57%)	4,949 (80.76)		650 (54.92%)	261 (43.57%)	398 (66.22%)	
Marital status	Yes	5,625 (83.19%)	537 (88.61%)	5,088 (82.65%)	<0.001	1,083 (89.36%)	537 (88.61%)	546 (90.10%)	0.402
	No	1,137 (16.81%)	69 (11.39%)	1,068 (17.35%)		129 (10.64%)	69 (11.39%)	60 (9.90%)	
Education	≤Elementary school	4,446 (65.75%)	141 (23.27%)	4,305 (69.93%)	<0.001	286 (23.60%)	141 (23.27%)	145 (23.93%)	0.960
	Middle school	1,418 (20.97%)	193 (31.85%)	1,225 (19.90%)		383 (31.60%)	193 (31.85%)	190 (31.35%)	
	≥High school	898 (13.28%)	272 (44.88)	626 (10.17%)		543 (44.80%)	272 (44.88%)	271 (44.72%)	
Health behavior variables									
Drinking	Yes	2,443 (36.13%)	296 (48.84%)	2,147 (34.88%)	<0.001	547 (45.13%)	296 (48.84%)	251 (41.42%)	0.009
	No	4,319 (63.87%)	310 (51.16%)	4,009 (65.12%)		665 (54.87%)	310 (51.16%)	355 (58.58%)	
Smoking	Yes	297 (8.11%)	40 (12.08%)	257 (7.72%)	0.006	69 (10.76%)	40 (12.08%)	29 (9.35%)	0.265
	No	3,364 (91.89%)	291 (87.92%)	3,073 (92.28%)		572 (89.24%)	291 (87.92%)	281 (90.65%)	
Health outcome variables									
Depression		13.84 ± 7.62	12.04 ± 5.74	14.02 ± 7.76	<0.001	12.32 ± 6.46	12.04 ± 5.76	12.60 ± 7.10	0.130
Health statement	Good	4,881 (72.20%)	526 (86.80%)	4,355 (70.77%)	<0.001	1,045 (86.22%)	526 (86.80%)	519 (85.64%)	0.560
	Not good	1,879 (27.80%)	80 (13.20%)	1,799 (29.23%)		167 (13.78%)	80 (13.20%)	87 (14.36%)	
IADL		0.61 ± 1.20	0.14 ± 0.54	0.66 ± 1.24	<0.001	0.14 ± 0.57	0.14 ± 0.54	0.14 ± 0.59	0.840
Chronic diseases	None	2,857 (42.25%)	262 (43.23%)	2,595 (42.15%)	0.084	530 (43.73%)	262 (43.23%)	268 (44.22%)	<0.001
	One kind	3,004 (44.42%)	283 (46.70%)	2,721 (44.20%)		436 (35.97%)	283 (46.70%)	153 (25.25%)	
	Two kinds	642 (0.49%)	41 (6.77%)	601 (9.76%)		168 (13.86%)	41 (6.77%)	127 (20.96%)	
	≥Three kinds	259 (3.83%)	20 (3.30%)	239 (3.88%)		78 (6.44%)	20 (3.30%)	58 (9.57%)	

Categorical variables are expressed as frequencies and percentages, and comparisons are made via the chi-square test or the exact probability method. Continuous variables are expressed as the means ± SDs and were compared via t tests or nonparametric tests, with *p* < 0.05 indicating statistical significance.

**Table 2:** Linear relationship between multidimensional internet use and cognitive ability.

Variables	Before PSM adjustment				After PSM adjustment			
	95%CI	*p*	Adjusted 95%CI	Adjusted *p*	95%CI	*p*	Adjusted 95%CI	Adjusted *p*
Internet use	0.182 (0.163, 0.201)	<0.001	0.059 (0.032, 0.086)	<0.001	0.100 (0.081, 0.119)	<0.001	0.079 (0.059, 0.099)	<0.001
Internet activities								
Chatting	0.177 (0.153, 0.202)	<0.001	0.068 (0.036, 0.100)	<0.001	0.074 (0.053, 0.096)	<0.001	0.061 (0.040, 0.083)	<0.001
Watching news	0.189 (0.168, 0.209)	<0.001	0.050 (0.021, 0.080)	0.001	0.101 (0.081, 0.120)	<0.001	0.079 (0.058, 0.100)	<0.001
Watching videos	0.177 (0.153, 0.200)	<0.001	0.048 (0.016, 0.081)	0.004	0.075 (0.054, 0.096)	<0.001	0.062 (0.041, 0.083)	<0.001
Playing games	0.182 (0.145, 0.218)	<0.001	0.041 (−0.07, 0.089)	0.095	0.071 (0.041, 0.101)	0.041	0.047 (0.017, 0.076)	0.002
Financial management	0.198 (0.128, 0.269)	<0.001	0.007 (−0.085, 0.099)	0.876	0.084 (0.029, 0.139)	0.003	0.044 (−0.012, 0.100)	0.121
Mobile pay	0.196 (0.166, 0.226)	<0.001	0.042 (0.003, 0.081)	0.037	0.092 (0.067, 0.116)	<0.001	0.068 (0.043, 0.093)	<0.001
WeChat use	0.182 (0.162, 0.202)	<0.001	0.062 (0.034, 0.090)	<0.001	0.094 (0.075, 0.114)	<0.001	0.076 (0.056, 0.100)	<0.001
Post WeChat moments	0.186 (0.163, 0.209)	<0.001	0.059 (0.027, 0.091)	<0.001	0.089 (0.068, 0.110)	<0.001	0.069 (0.048, 0.090)	<0.001
Internet use of equipment								
Desktop computers	0.201 (0.160, 0.241)	<0.001	0.024 (−0.032, 0.080)	0.406	0.091 (0.058, 0.123)	<0.001	0.061 (0.028, 0.093)	<0.001
Laptops computers	0.205 (0.126, 0.285)	<0.001	0.033 (−0.063, 0.130)	0.502	0.091 (0.029, 0.153)	0.004	0.062 (0.003, 0.122)	0.041
Tablets computers	0.175 (0.111, 0.240)	<0.001	0.036 (−0.037, 0.109)	0.337	0.061 (0.010, 0.112)	0.019	0.037 (−0.013, 0.089)	0.147
Mobile phone	0.181 (0.161, 0.201)	<0.001	0.061 (0.033, 0.089)	<0.001	0.093 (0.073, 0.113)	<0.001	0.073 (0.053, 0.093)	<0.001
Internet use of frequency								
Not regularly	0.106 (0.032, 0.180)	0.005	0.022 (−0.082, 0.126)	0.678	−0.011 (−0.069, 0.047)	0.719	−0.006 (−0.063, 0.052)	0.850
Almost every week	0.083 (0.050, 0.117)	<0.001	0.009 (−0.041, 0.058)	0.729	0.026 (−0.001, 0.052)	0.055	0.017 (−0.010, 0.042)	0.206
Almost every day	0.061 (0.055, 0.068)	<0.001	0.020 (0.011, 0.030)	<0.001	0.032 (0.025, 0.038)	<0.001	0.025 (0.018, 0.032)	<0.001

Linear regression models were introduced with *p* values adjusted for baseline characteristics (age, urban/rural, alcohol use, and chronic disease) to exclude potential bias in the results due to age, urban/rural, alcohol use, and chronic disease, with *p* < 0.05 indicating a statistically significant difference. 95%CI: 95% confidence interval of the odds ratio.

### 2.2 Measurements

This study examines cognitive function as the primary outcome, measured by the Chinese version of the Mini-Mental State Examination (MMSE) in the CHARLS survey (22, 23). The MMSE was used to measure the subjects’ overall cognitive function (24). This screening test comprises 30 items that assess various cognitive domains, including naming, attention, calculation, abstraction, orientation, memory, visual–spatial skills, and language function. In this study, the scale was divided into five dimensions: language ability (9 points), attention and calculation (5 points), orientation (10 points), memory (3 points), and recall (3 points), totaling 30 points. A lower total score indicates poorer cognitive function.

MIU was the primary independent variable. The following questions were used to measure it in the CHARLS questionnaire (Version 2018): (1) Have you used the internet in the last month? (2) If so, what types of devices would you use to access the internet? (3) How often did you use the internet in the last month? (4) What do you usually do on the internet? (5) Do you use mobile payment methods, such as Alipay or WeChat Pay? (6) Do you use WeChat? (7) Do you post WeChat Moments?

The control variables included demographic factors (gender, age, education, marital status, residency), health behaviors (drinking and smoking), and health outcomes (depressive symptoms, self-reported health, IADL, and chronic disease status). These variables were included to account for factors that may influence cognitive function and internet usage among the study population.

### 2.3 Statistical analysis

Statistical analyses were conducted using Stata. Propensity score matching (PSM) was performed based on internet use, with a 1:1 matching ratio. The cognitive ability scores were logarithmically transformed due to significant differences in the values of the cognitive ability variables. Descriptive statistics were then performed on the covariates, with differences between the internet-use and non-internet-use groups analyzed using chi-square tests or t-tests. Next, we conducted a regression analysis to examine the relationship between MIU and cognitive ability in older adults, adjusting for baseline imbalances caused by certain factors, which were included in the regression model to calculate the adjusted *p*-value. Finally, multiple linear regression analysis was conducted to explore the associations between internet use and the components of cognitive ability.

In the robustness test, mobile phones were used to replace the explanatory variables to revalidate the relationship between internet use and cognitive ability. Owing to the impact possibly varying among different ages of older adults, they were divided into low (60–74 years old) and high (≥75 years old) age groups.

## 3 Results

### 3.1 Descriptive analysis

After PSM, the online and nononline groups were balanced in terms of sex, marital status, education, smoking status, depression status, and self-rated health; however, there were still significant differences in terms of age, urban/rural area, drinking status, IADL and chronic diseases. In the paired sample (*n* = 1,212), the mean age was 66.36 ± 5.66 years, and 436 (35.97%) patients had a chronic disease. In terms of age, the internet-use group was younger than the non-Internet-use group (65.90 years and 66.82 years, respectively). Additionally, the majority of the internet-use group resided in urban areas (338, 56.43%), while the majority of the non-internet-use group was from rural areas (203, 33.78%).

### 3.2 Associations between MIU and cognitive ability

It was not difficult to determine whether online behavior positively affected cognitive ability after PSM matching. In addition to financial management, there was an optimistic impact on the cognitive level (*p* < 0.001). The use of devices such as desktop computers, laptops, and mobile phones was also associated with improved cognitive ability (*p* < 0.001). Notably, frequent internet access—almost daily—had a significant impact on the cognitive levels of older adults (*p* < 0.001). To further explore which part of cognitive ability was affected by internet activities, Table 3 presents a breakdown of cognitive ability into five components. While internet use had no effect on recall (*p* = 0.587), it significantly impacted language ability, attention and calculation, orientation, and memory (*p* < 0.001).

**Table 3:** Effects of internet use on various components of cognitive ability in older adults.

Variables	Before PSM adjustment				After PSM adjustment			
	95%CI	*p*	Adjusted 95%CI	Adjusted *p*	95%CI	*p*	Adjusted 95%CI	Adjusted *p*
Language ability	0.251 (0.222, 0.280)	<0.001	0.075 (0.033, 0.116)	<0.001	0.130 (0.101, 0.160)	<0.001	0.094 (0.064, 0.124)	<0.001
Attention and calculation ability	0.304 (0.247, 0.361)	<0.001	0.173 (0.089, 0.256)	<0.001	0.182 (0.109, 0.255)	<0.001	0.175 (0.100, 0.251)	<0.001
Orientation	0.123 (0.105, 0.142)	<0.001	0.025 (−0.003, 0.053)	0.083	0.069 (0.051, 0.088)	<0.001	0.049 (0.030, 0.067)	<0.001
Memory	0.118 (0.091, 0.146)	<0.001	0.032 (−0.008, 0.072)	0.118	0.059 (0.031, 0.088)	<0.001	0.036 (0.006, 0.066)	0.017
Recall	0.41 (0.009, 0.074)	0.013	0.009 (−0.040, 0.058)	0.724	0.011 (−0.030, 0.053)	0.594	0.012 (−0.032, 0.056)	0.587

Linear regression models were introduced with *p* values adjusted for baseline characteristics (age, urban/rural, alcohol use, and chronic disease) to exclude potential bias in the results due to age, urban/rural, drinking, and chronic disease, with *p* < 0.05 indicating a statistically significant difference. 95%CI: 95% confidence interval of the odds ratio.

To further test the impact of internet use on the physical health of older adults, this study adopted the method of replacing variables to conduct a robustness test. Given that 90.43% of older adults use mobile phones for online activities, this study replaced mobile phone use with an explanatory variable to assess the robustness of the model. The results were found to be statistically significant (Supplementary Table S1).

### 3.3 Associations between MIU and cognitive ability in the PSM-matched 60–75 and ≥75 age groups

On the basis of the above results, considering that age may affect the relationship between internet use and cognition, this study further explored the relationship between internet behavior and various components of cognitive ability in older adults of different ages. Older adults were categorized into two age groups: 60–75 years and ≥75 years. These results are consistent with those of previous studies (activities *β* = 0.017 [0.012, 0.022]; devices *β* = 0.055 [0.039, 0.071]; frequency *β* = 0.029 [0.021, 0.036]), further supporting the results of this study. However, it is noteworthy that in the older age group, playing games as an internet activity and desktop computers showed no significant association with cognitive level, whereas the rest remained the same (Supplementary Table S2).

## 4 Discussion

The analysis revealed that MIU played a positive role in improving the cognitive level of older adults. It was found that the components of cognitive ability—language, attention and calculation, orientation, and memory—were positively correlated with MIU. Additionally, when the older adult population was divided into two age groups, the impact of MIU on cognitive improvement was more pronounced in the younger age group (60–75 years old).

First, there was a significant positive correlation between MIU and cognitive ability among older adults. In addition, the results of the robustness test were consistent with the results of the baseline regression of this study, confirming the robustness and credibility of MIU’s cognitive enhancement effect on older adult individuals. Regarding internet activities, online social interactions—such as chatting, using WeChat, and posting to friends—play a crucial role in mental development. Engaging in complex social environments is essential for the normal development of the human mind (25). These social interactions help increase “cognitive reserve”—the brain’s flexible and effective use of cognitive networks—which enables people to continue performing cognitive tasks despite changes in the brain (26). Additionally, social engagement can improve cognitive functioning in older adults by expanding their social networks and increasing emotional support (27). Second, watching news and videos provides older adults with a valuable source of information. This combination of visual and auditory input fully engages their senses and stimulates the brain. Exposure to varied, sometimes conflicting information also helps keep their understanding of the world up-to-date (27). Moreover, playing games may help slow cognitive decline in older adults, possibly because the central nervous system retains some plasticity even later in life (28). The underlying mechanism may be that games require quick responses within a short timeframe, which helps older adults maintain their awareness of the external world and enhances their cognitive abilities (29). Notably, financial management activities offer fewer cognitive benefits than online activities, likely due to the more conservative approach of older adults toward finances.

Additionally, the positive impact of using desktop computers and laptops on cognitive ability in older adults is consistent with previous studies. For instance, infrequent computer use has been associated with smaller hippocampal volumes, which are linked to cognitive function, in cognitively healthy older adults (16). Notably, mobile phone usage is significantly higher among older adults than computers. This trend supports earlier findings that the lightweight, user-friendly design of mobile phones makes them more accessible and convenient for older users (30). Higher usage of mobile devices further strengthens the cognitive benefits of online activities for this age group. Finally, some studies suggest a U-shaped relationship between the frequency of internet use and the risk of cognitive impairment, where both very low and very high usage could be linked to cognitive challenges (13). However, owing to the lack of detailed data on daily mobile phone usage in the CHARLS database, this study was unable to determine the specific hours that might indicate excessive computer use. More research is needed to clarify this area.

Internet use profoundly impacts the cognitive faculties of older adults, influencing language, attention, calculation, orientation, memory. A decline in language ability hampers social engagement and independence, making its preservation vital for maintaining dignity and social ties. Enhanced physical fitness is associated with a slower decline in language skills during aging. A longitudinal study indicated that impaired attention is a key factor in determining quality of life for those with cognitive impairments (31, 32). Internet use can aid in maintaining numeracy skills. Furthermore, disorientation is frequently associated with memory decline, which impairs an individual’s ability to remain oriented to time and surroundings (33). The absence of a dedicated companion can jeopardize older adults’ personal safety. Finally, memory retention allows older adults to adjust to a changing world, reducing the sense of isolation often caused by declining learning ability.

Aging diminishes the relationship between internet use and cognitive ability. This decline aligns with the morphology theory of intelligence, which differentiates between crystalline and fluid intelligence. Fluid intelligence, crucial for acquiring new knowledge and solving problems, typically peaks around age 20 and declines with age (34). As a relatively new technology, the internet demands considerable fluid intelligence. By age 75, individuals typically face an increased risk of cognitive decline. This cognitive decline not only impairs their abilities but also reduces the internet’s positive impact on their lives. Empirical studies confirm that age 75 marks a significant threshold, with Wei-Ju Le noting that the prevalence of cognitive disorders rises sharply to 22.7% in individuals over 75, compared to 2.2% in those aged 53–64 and 10.2% in those aged 65–74 (35). These figures highlight the critical need to address cognitive health issues as people age, particularly in the context of technology use.

This study makes both theoretical and practical contributions to the literature. First, it provides preliminary evidence in the Chinese context, where internet use among older adults is still at an early stage compared to developed countries. Second, the study highlights the internet’s potential as an assistive tool for older adults in the digital age. Several steps were taken to ensure the robustness of the findings. On one hand, the use of propensity score matching (PSM) largely controls for confounding factors between the two study groups; on the other hand, the positive impact of internet use on cognitive ability was further validated by substituting explanatory variables.

Certain limitations should also be acknowledged. First, given the observational and cross-sectional design of this study, inferring causal relationships was not feasible. Second, the presence of unmeasured covariates may have introduced confounding bias, despite accounting for various confounding factors. Third, the data were collected through self-administered questionnaires, which could have led to recall errors and may not accurately reflect the actual frequency of internet use. Due to the lack of specific timing data on internet usage in the CHARLS database, it was not possible to further investigate the impact of specific internet usage durations on the cognitive abilities of older adults.

## 5 Conclusion

This study offers preliminary evidence that MIUs are positively linked to cognitive performance, with all seven internet activities contributing to cognitive enhancement. Specifically, the use of mobile phones, desktops, and laptops was found to positively influence cognitive ability, while daily internet use proved especially impactful. The subgroup analysis further revealed that aging diminishes the strength of the relationship between internet use and cognitive function. Future studies should examine the long-term effects of internet use on cognitive health in diverse populations. Moreover, policies aimed at improving internet accessibility and fostering digital literacy among the older adult could help mitigate cognitive health disparities.

## Data Availability

The datasets presented in this study can be found in online repositories. The names of the repository/repositories and accession number(s) can be found in the article/Supplementary material.
