# Supplementary material for: Multidimensional internet use related to cognitive performance in older persons: a nationwide cross-sectional study
Source: Front Public Health. 2024 Dec 16;12:1492331. doi: 10.3389/fpubh.2024.1492331 (PMC11683067; doi:10.3389/fpubh.2024.1492331)
Supplement: Supplementary file 1 [file Table_1.docx]

**Supplementary Table S1**  Linear relationship between multidimensional internet use and cognitive ability

| Variables | Before PSM adjustment | | | | After PSM adjustment | | | |
| --- | --- | --- | --- | --- | --- | --- | --- | --- |
|  | 95%CI | P | Adjusted 95%CI | Adjusted P | 95%CI | P | Adjusted 95%CI | Adjusted P |
| Mobile phone | 0.181  (0.161,0.201) | ＜0.001 | 0.061  (0.033,0.089) | ＜0.001 | 0.093  (0.073,0.113) | ＜0.001 | 0.073  (0.053,0.093) | ＜0.001 |

Note: Linear regression models were introduced with P values adjusted for baseline characteristics (age, urban/rural, alcohol use, and chronic disease) to exclude potential bias in the results due to age, urban/rural, drinking, and chronic disease, with P<0.05 indicating a statistically significant difference. 95% CI: 95% Confidence Interval of the Odds Ratio.

**Supplementary Table S2** Linear relationship between multidimensional internet use and cognitive ability in the PSM-Matched 60-75 and ≥75 Age Groups

| Before PSM adjustment | | | | | After PSM adjustment | | | |
| --- | --- | --- | --- | --- | --- | --- | --- | --- |
| Variables | 60-74岁  95%CI | P | Adjusted  95%CI | Adjusted P | ≥75岁  95%CI | P | Adjusted  95%CI | Adjusted P |
| Internet use | 0.105  (0.084,0.126) | ＜0.001 | 0.092  (0.073,0.112) | ＜0.001 | 0.065  (0.011,0.119) | 0.018 | 0.070  (0.015,0.125) | 0.013 |
| Internet activities | 0.021  (0.016,0.025) | ＜0.001 | 0.017  (0.012,0.022) | ＜0.001 | 0.022  (0.013,0.031) | ＜0.001 | 0.016  (0.006,0.025) | ＜0.001 |
| Chating | 0.077  (0.054，0.100) | ＜0.001 | 0.071  (0.050,0.092) | ＜0.001 | 0.048  (-0.016,0.111) | 0.140 | 0.067  (0.006,0.129) | 0.033 |
| Watching news | 0.103  (0.082,0.125) | ＜0.001 | 0.078  (0.058,0.098) | ＜0.001 | 0.080  (0.025,0.135) | 0.005 | 0.075  (0.020,0.130) | 0.008 |
| Watching videos | 0.076  (0.053,0.098) | ＜0.001 | 0.064  (0.043,0.084) | ＜0.001 | 0.071  (0.010,0.132) | 0.023 | 0.071  (0.012,0.130) | 0.019 |
| Playing games | 0.072  (0.040,0.104) | ＜0.001 | 0.044  (0.014,0.073) | 0.004 | 0.058  (-0.031,0.147) | 0.199 | 0.062  (-0.028,0.152) | 0.177 |
| Financial management | 0.082  (0.023,0.140) | 0.007 | 0.038  (-0.017,0.092) | 0.180 | 0.111  (-0.049,0.271) | 0.173 | 0.056  (-0.094,0.205) | 0.462 |
| Mobile pay | 0.095  (0.069,0.122) | ＜0.001 | 0.066  (0.042,0.091) | ＜0.001 | 0.049  (-0.038,0.135) | 0.268 | 0.027  (-0.054,0.108) | 0.514 |
| Wechat use | 0.098  (0.077,0.120) | ＜0.001 | 0.089  (0.067,0.106) | ＜0.001 | 0.065  (0.008,0.122) | 0.025 | 0.073  (0.018,0.128) | 0.010 |
| Post WeChat moments | 0.093  (0.070,0.115) | ＜0.001 | 0.073  (0.052,0.093) | ＜0.001 | 0.056  (-0.007,0.119) | 0.083 | 0.0667  (0.005,0.130) | 0.035 |
| Internet use of devices | 0.069  (0.054,0.085) | ＜0.001 | 0.055  (0.039,0.071) | ＜0.001 | 0.068  (0.042,0.095) | ＜0.001 | 0.048  (0.020,0.075) | ＜0.001 |
| Desktop computers | 0.096  (0.061,0.132) | ＜0.001 | 0.053  (0.020,0.086) | 0.002 | 0.054  (-0.027,0.136) | 0.189 | -0.001  (-0.080,0.078) | 0.977 |
| Laptops computers | 0.093  (0.026,0.160) | 0.006 | 0.045  (-0.015,0.106) | 0.144 | 0.076  (-0.084,0.237) | 0.348 | 0.062  (-0.087,0.210) | 0.413 |
| Tablets computers | 0.069  (0.010,0.129) | 0.022 | 0.049  (-0.005,0.103) | 0.075 | 0.038  (-0.054,0.130) | 0.413 | 0.006  (-0.083,0.095) | 0.895 |
| Mobile phone | 0.097  (0.076，0.118) | ＜0.001 | 0.085  (0.066,0.105) | ＜0.001 | 0.068  (0.009，0.124) | 0.023 | 0.076  (0.021,0.132) | 0.007 |
| Internet use of frequency | 0.035  (0.027,0.043) | ＜0.001 | 0.029  (0.021,0.036) | ＜0.001 | 0.039  (0.024,0.053) | ＜0.001 | 0.028  (0.013,0.043) | ＜0.001 |
| Not regularly | -0.015  (-0.076,0.046) | 0.627 | -0.003  (-0.058,0.052) | 0.912 | 0.063  (-0.162,0.289) | 0.579 | 0.126  (-0.168,0.420) | 0.396 |
| Almost every week | 0.055  (-0.001,0.111) | 0.056 | 0.039  (-0.011,0.089) | 0.129 | 0.020  (-0.141,0.180) | 0.811 | 0.016  (-0.155,0.187) | 0.856 |
| Almost every day | 0.100  (0.079,0.121) | ＜0.001 | 0.087  (0.067,0.107) | ＜0.001 | 0.062  (0.007,0.118) | 0.028 | 0.064  (0.009,0.119) | 0.023 |

Note: Linear regression models were introduced with P values adjusted for baseline characteristics (age, urban/rural, alcohol use, and chronic disease) to exclude potential bias in the results due to

age, urban/rural, drinking, and chronic disease, with P<0.05 indicating a statistically significant difference. 95% CI: 95% Confidence Interval of the Odds Ratio
